# A genetic linkage map and improved genome assembly of the termite symbiont *Termitomyces cryptogamus*

**DOI:** 10.1186/s12864-023-09210-x

**Published:** 2023-03-16

**Authors:** Sabine M. E. Vreeburg, Ben Auxier, Bas Jacobs, Peter M. Bourke, Joost van den Heuvel, Bas J. Zwaan, Duur K. Aanen

**Affiliations:** 1 Laboratory of Genetics, Wageningen University & Research, Wageningen, the Netherlands; 2 Biometris, Wageningen University & Research, Wageningen, the Netherlands; 3 Plant Breeding, Wageningen University & Research, Wageningen, the Netherlands

**Keywords:** GBS, Recombination, Basidiomycete, Mutualistic symbiosis

## Abstract

**Background:**

The termite-fungus symbiosis is an ancient stable mutualism of two partners that reproduce and disperse independently. With the founding of each termite colony the symbiotic association must be re-established with a new fungus partner. Complementarity in the ability to break down plant substrate may help to stabilize this symbiosis despite horizontal symbiont transmission. An alternative, non-exclusive, hypothesis is that a reduced rate of evolution may contribute to stabilize the symbiosis, the so-called Red King Effect.

**Methods:**

To explore this concept, we produced the first linkage map of a species of *Termitomyces*, using genotyping by sequencing (GBS) of 88 homokaryotic offspring. We constructed a highly contiguous genome assembly using PacBio data and a de-novo evidence-based annotation. This improved genome assembly and linkage map allowed for examination of the recombination landscape and its potential effect on the mutualistic lifestyle.

**Results:**

Our linkage map resulted in a genome-wide recombination rate of 22 cM/Mb, lower than that of other related fungi. However, the total map length of 1370 cM was similar to that of other related fungi.

**Conclusions:**

The apparently decreased rate of recombination is primarily due to genome expansion of islands of gene-poor repetitive sequences. This study highlights the importance of inclusion of genomic context in cross-species comparisons of recombination rate.

**Supplementary Information:**

The online version contains supplementary material available at 10.1186/s12864-023-09210-x.

## Introduction

An emblematic mutualism that impacts many tropical ecosystems is the mutualistic symbiosis between *Termitomyces* fungi and fungus-farming termites of the family Macrotermitinae [1]. This obligate mutualism has recognized roles as engineers of ecosystems [2], major decomposers of the African savannahs [3], and agricultural pests [4]. In addition, the mushrooms seasonally produced by the fungus are considered local delicacies that contain important nutrients, including high amounts of essential amino acids [5–8]. In the areas where *Termitomyces* is found, its mushrooms are considered to be an important source of nutrition and income [9].

The termite-fungus symbiosis is an example of a symbiotic mutualism that has remained stable over 30 million years [10, 11], despite horizontal transmission of the fungal symbiont [12–14]. Horizontal transmission means that reproduction of the host and symbiont are not coupled, but instead each new generation needs to re-establish the interaction. Theory indicates that horizontal transmission does not necessarily fully align the reproductive interests of host and symbiont, and this produces an evolutionary tension [15]. An association of multiple genetically unrelated fungal symbionts with a single host may have direct or indirect costs due to selection for antagonistic traits that have negative side effects for the host. Therefore, the host is under selection to reduce symbiont dispersal and the associated mixing between genetically different symbionts [16]. This conflict is reduced through positive frequency dependant selection that results in maintenance of a single fungal symbiont inside a termite colony [17].

At higher taxonomic levels there is some phylogenetic specificity in the fungus-termite associations; clades within *Termitomyces* associate with certain termite genera, although there is little specificity at finer taxonomic levels [10, 18]. The factors underlying the observed interaction specificities are currently unknown, although it has been hypothesized that differing complementarities in substrate breakdown may be involved [19–22]. This was further explored using genomes of the three symbionts; the fungus *Termitomyces cryptogamus*, the termite *Macrotermes natalensis,* and termite gut microbiota [23, 24]. Analysis of the termite and fungal partners showed a level of complementarity in the enzymatic machinery for processing the complex and variable substrate that they feed on, and this was hypothesized to be one factor ensuring faithfulness and enhancing the stability of the symbiosis.

In most species of fungus-growing termites, symbiont acquisition involves sexual reproduction of the fungal symbiont through the formation of mushroom fruiting bodies [13]. However, within a termite colony a single fungal individual is continuously vegetatively propagated [17]. After the reproductive termite class, alates, disperses from a colony, the fruiting bodies produce sexual spores that are spread into the environment, inferred to function for inoculation of newly founded colonies [9, 12, 13, 20, 25, 26]. Species of *Termitomyces* have never been found to occur outside of a termite colony, and presumably spend most, if not all, of their lives inside a single termite colony [1]. In such stable biotic environments the process of sex, defined as any process involved in reshuffling chromosomal genomes, may be selected against [27]. Additionally, mutualistic interactions, once evolved favour reduced rates of evolution, the so-called Red King Effect, in contrast to the rapid evolution found in antagonistic relationships [28]. While independent segregation of chromosomes is a necessary by-product of the single crossover necessary for ploidy maintenance, the placement of these crossovers does not necessarily lead to recombination within a chromosome [29, 30]. Telomeric recombination, as seen in *Agaricus*, limits the recombination in the sexual cycle to the assortment of chromosomes [30].

To study recombination, high-quality contiguous reference assemblies are needed. The emergence and further development of sequencing technologies have revolutionised genetic research. Genomes are now available for many non-model species; however, many of these are based on short-read technologies, which cannot process repetitive regions of the genome resulting in highly fragmented assemblies (e.g. [24]). Recent evidence suggests that these repetitive regions are more relevant than previously recognized, but research is hampered by a lack of contiguous reference assemblies [31]. Such assemblies also facilitate genetic mapping studies, as well as analyses on biosynthetic gene clusters and synteny [31, 32].

Here, we focused on the study of recombination in *T*. *cryptogamus.* We first produced an improved *T. cryptogamus* reference assembly using long-read sequencing technology. Subsequently, we constructed the first genetic linkage map of a *Termitomyces* species using markers generated by Genotyping by Sequencing (GBS) of single-spore cultures from a fruiting body of *T. cryptogamus* harvested from a *Macrotermes natalensis* termite colony. Finally, to analyse the recombination landscape of *Termitomyces cryptogamus*, we used the positional information of the SNP markers on the v2.0 assembly to compare the physical to the genetic distance. We then compared the rate of recombination to other published values of both domesticated and free-living species.

## Materials and methods

### Strains and culture conditions

For the improved assembly the same homokaryotic strain of *T. cryptogamus* P5 was used as for the original genome assembly [24]. While the original publication referred to this species as *Termitomyces* sp., it has recently been described as *T. cryptogamus* [33]. The fungus was grown in liquid culture in Malt Yeast (MY) medium (20 g malt, 2 g yeast, 1 L demineralized water) and mycelium was lyophilized.

For linkage mapping, a population of homokaryotic siblings was obtained from fruiting bodies of a single *Macrotermes natalensis* colony in Modimolle, South Africa (GPS: S24 40.484 E28 48.271). Mushroom fruiting bodies of *T. cryptogamus* were obtained by incubation of fungal combs [34]. Spores were deposited on Petri dishes containing Malt Yeast Agar (MYA: 20 g malt, 2 g yeast, 15 g Agar, 1 L demineralised water) for a range of time periods varying from a few seconds up to 24 hours. Petri dishes with spore prints were incubated at 25 °C and monitored daily for hyphal growth under a dissecting microscope. To isolate homokaryons, small patches of hyphal growth were transferred onto fresh MYA using a needle.

### Homokaryotic basidiospore colony screening

As random sexual spore deposition on the petri plates can allow for sibling matings, it was necessary to distinguish unmated homokaryons from mated heterokaryons. Heterokaryons of *Termitomyces* have been shown to have higher growth rates than homokaryons, enabling a rough visual selection based on growth rate [35]. To test our categorization of homokaryons and heterokaryons, we developed a Restriction Fragment Length Polymorphism (RFLP) marker in a highly variable intron of the nuclear Elongation Factor 1 alpha gene using primers EF595F and EF1160R [20] and subsequent Sanger Sequencing of the PCR product of the parental heterokaryon. This revealed a polymorphism surrounding a NdeI restriction site.

### Isolation, sequencing, and genome assembly of a parental homokaryon, mt50a

To recover the haploid genomes from the parental heterokaryon, allowing reconstruction of marker phasing, we prepared protoplasts as previously described [35]. The regenerated protoplasts were expected to be a mixture of heterokaryotic and homokaryotic cultures and were analysed with the RFLP marker described above. Recovered homokaryotic cultures were of one parental genotype, the allele without the NdeI restriction site. One homokaryon isolated, designated mt50a, was used for DNA extraction, and Illumina paired-end reads were generated. Reads were assembled using SPAdes [36].

### DNA isolation and sequencing

For the subsequent PacBio sequencing of the P5 homokaryon, DNA was extracted using a CTAB protocol as described previously [37]. Removal of RNA, library preparation and sequencing were performed by BGI Genomics. Sequencing was performed using the PacBio Sequel System.

For extraction of linkage mapping progeny, and the parental homokaryon mt50a described above, cultures were grown in liquid MY medium. DNA was isolated using a CTAB extraction. RNA present in the samples after extraction was degraded by 3 μl RNase I (Thermo Scientific) incubated for 2 h at 37 °C. Genotyping-by-sequencing (GBS) GBS was performed at the Genomic Diversity Facility of Cornell University, using a previously described protocol [38]. The enzyme used for the restriction step was EcoT22I (a six-base cutter) rather than ApeKI (a five-base cutter with one wobble base).

### Assembly of the *Termitomyce*s *cryptogamus* v2.0 genome

To produce an updated genome assembly PacBio subreads of *T. cryptogamus* P5 were assembled using the CANU assembler v1.7 [39] using the estimated genome size of Poulsen et al. (83.7 Mb) for the parameter “genomeSize”. Previously published Illumina data of strain P5 [24] was obtained from the NCBI databases and trimmed for quality in CLC Genomics Workbench v9 using default settings. The trimmed reads were used for two rounds of polishing of the CANU assembly using Pilon v1.22 [40] with default settings. Genome statistics were obtained by QUAST v4.6.3. (−m 0 to be able to compare to v1.0 genome statistics) [24, 41] and assembly completeness was assessed using BUSCO v3, gene set Basidiomycota odb9 [42]. The PacBio subreads were then aligned to the assembly using minimap2 v2.17-r954-dirty [43] and average alignment depth was calculated using samtools v1.7 [44]. The assembly was aligned to the previous version (v1.0) of the reference genome of *Termitomyces* [24], as well as one of the parental homokaryons (see below) using minimap2 v2.17 [43]. These alignments were visualised using dotPlotly (v1.0:v2.0 comparison -q 1000, −m 1000; mt50a:v2.0 comparison -q500 -m500) (https://github.com/tpoorten/dotPlotly). The mitochondrion contig was manually identified using BLAST based on a previous published mitochondrial sequence [37].

### RNA isolation and sequencing

To assist with gene prediction and annotation of the v2.0 genome, RNA was obtained from laboratory cultivation of additional *Termitomyces cryptogamus* strains T153 and T112, both also isolated from South Africa, after growing on Potato Dextrose Agar (Difco™ 39 g/L) for 10 days at room temperature. Mycelium was harvested by scraping it from agar plates with a scalpel, freezing it in liquid nitrogen and storing it at − 80 °C until RNA extraction. RNA was extracted by grinding frozen mycelium to a fine powder under liquid nitrogen and using the Isolate II RNA Plant Kit (Bioline) according to the manufacturer’s protocol. RNA extracts underwent 100 bp paired-end BGISeq-500 sequencing.

For extraction of RNA from the parental heterokaryon Mn132, the parent of the mapping population used here, 20 mg of nodules, fresh comb, or old comb was frozen in liquid nitrogen and ground with a pestle to a fine powder. RNA was isolated using the RNeasy plant minikit (Qiagen, Hilden, Germany), according to the manufacturer’s protocol. After RNA purity and quality were determined in a NanoDrop spectrophotometer (Thermo Scientific, Wilmington, DE, USA) and RNA yield and integrity analyses in Experion (Bio-Rad Laboratories, Hercules, CA, USA) RNA was enriched by oligo(dT) beads to construct cDNA libraries, which were subsequently sequenced with 125 bp paired-end reads on the Illumina HiSeq 2500 platform.

### Linkage map generation

The resulting GBS reads of the mapping population were demultiplexed using sabre version 1.000 and, together with the raw reads of the paretal homokaryon mt50a, aligned to the v2.0 genome using the BWA-MEM algorithm v0.7.17 [45]. SNPs were called using Freebayes v1.3.1 [46] with a minimum read depth (−C) of 5.

For strict filtering of the VCF file the TASSEL GUI v 5.2.58 was used [47]. We initially filtered by position: min individuals called = 40, min allele frequency (AF) = 0.2, max AF = 0.8, max heterozygous calls = 0.1. Then, to filter out heterokaryons, we filtered out all samples with more than 10% heterozygous SNPs. Subsequently, we repeated the first filter by position, set all heterozygous calls to unknown and removed the blank control. Next, we removed all positions with three alleles, all positions that were missing in the mt50a parent, all positions with more than 10% missing and the positions with a cumulative *p*-value of the observed minor allele frequency < 0.01 (under a binomial distribution). Then we removed those positions that had SNP calls that were not supported by adjacent markers, i.e. SNP calls that would require a double crossover. Finally, we used the R package polymapR v.1.0.20 [48] to filter out positions with the function checkF1 (qall_weights < 0.75), individuals with more than 10% missing positions and positions that had less than 5 unique markers in a linkage group at LOD > 5.5.

The linkage map was constructed using the R package polymapR in Rstudio v3.6.1, with a dummy “nulliplex” parent added, to make it suitable for our mono-parental mapping population (analogous to a back-cross population with a completely homozygous recurrent parent). Linkage groups were assigned with a LOD score > 5.5. The resulting linkage map was visualised using the R package LinkageMapView [49].

### Mapping of the mating type locus

Matings were set up using 30 individuals of our mapping population on Petri dishes as described previously [35], using morphology to assess mating success. Six of the siblings were crossed against all siblings (Supplementary Table 1). To further clarify growth characteristics, a piece of mycelium was transferred from both homokaryons and the interaction zone of each mating (Supplementary Fig. 1A + B). The phenotypic data were added as a binary trait (0/1) to the strictly filtered dataset and linkage mapping was performed as described above to map the mating type locus. The mating type locus was added to the map after making the core map with strictly filtered data, as it was scored for only the 30 individuals.

To confirm the resulting map position of the mating-type locus, the predicted HD1 and HD2 proteins from *Schizophyllum commune* H4-8 (XP_003038830.1 and XP_003037496.1 respectively) were used as queries against the genome using TBLASTN 2.9.0+ [50]. To find the putative location of the pheromone/pheromone-receptor, the *S. cerevisiae* pheromone receptor Ste3 (sp|P06783) was used as a query. Conserved domains of genes surrounding matches were identified using the NCBI Conserved Domain Database [51].

### Recombination frequency

To identify recombination events, we removed all individuals that the previous filter indicated as heterokaryons, and then filtered by position (minimum individuals called = 10, minor allele frequency (AF) = 0.05). Then we removed all positions that had a heterozygous call for the mt50a parent and all positions that had more than one heterozygous call over all samples. We changed all remaining heterozygous calls to missing. We retained only biallelic sites and removed positions with more than 30 missing calls. We manually curated the marker set by inspecting the alignments of suspect markers in IGV [52]. In this dataset 180 extra markers were included, that had a minor allele frequency with an expected cumulative *p*-value < 0.01 under a binominal distribution that had been filtered out in the strictly filtered dataset.

### Forced order linkage map

To compare the linkage map data against the highly contiguous PacBio assembly, we used the linkage function of polymapR [48] to calculate the extent of linkage between the first two and last two markers of each scaffold in the less strictly filtered genotyping data. We connected all contigs of which the most distal markers were linked with a LOD score above 5.5. We imputed missing values by replacing any missing data point with the previous allele, unless it was at the start of a contig, in which case we used the next data point. We calculated recombination frequency to obtain a forced order linkage map and compared the linkage groups to the linkage groups of the linkage map that was constructed with the strictly filtered dataset using the function compare_maps of polymapR.

### Comparison physical and genetic distance in the forced order map

To examine the relation between physical and genetic distance, genetic positions were plotted against physical positions for all linkage groups in the forced-order linkage map. To assess whether there were intervals with more or less recombination compared to the average number of crossovers, we first examined whether there was a correlation between the number of crossovers in an interval between markers and the number of recombination events between those markers. We then constructed a probability distribution, given the marker distance, i.e. the probability of finding a recombination event in the mapping population between two markers is equal to the marker distances between these two markers divided by the sum of all marker distances in our map. Then we drew 10,000 multinominal random samples, with probability as explained above. For each interval between two markers, we compared the number of crossovers in our population to all random samples and calculated the probability of finding more or fewer crossovers than the observed number of crossovers. We corrected the obtained *p*-values for multiple testing using a Benjamini-Hochberg procedure [53].

### Annotation of genome v2.0

Functional annotation of the genome assembly was performed using the funannotate pipeline [54]. Genes were predicted using a hybrid approach combing RNA-seq and de-novo predictions. RNA-seq data from field samples of *T. cryptogamus* was used to inform the de-novo annotation. Briefly, RNA-seq samples were collected as described above and combined to assemble a de-novo transcriptome using trinity, and the assembled transcripts mapped to the genome using minimap2 [43]. Genes were predicted using a combination of Augustus, GlimmerHMM, and CodingQuarry [55–57]. Predicted genes were functionally annotated using EGGnog, InterProScan and dbCAN2 databases [58–60].

To assess potential centromere locations, average GC% and gene density was calculated using bedtools 2.26.0 [61] in 30 kb overlapping windows, with a sliding step of 10 kb. To assess gene spacing, intergenic spaces were extracted from the genome annotation also using bedtools, excluding 5′ and 3′ untranslated regions by filter GFF entries using the keyword “gene”. To compare against other fungi, genomes and annotations were obtained from the NCBI databases and processed in the same manner. The genomes used were: *Coprinopsis cinereus* (Psyathrellaceae) GCA_014872705.1 [62], *Schizophyllum commune* (Schizophyllaceae) GCF_000143185.1 [63], *Agaricus bisporus* (Agaricaceae) GCA_014872705.1 [30], *Hypsizygus marmoreus* (Lyophyllaceae) GCA_013433165.1 [64] and *Armillaria ostoyae* (Physalacriaceae) LR732075 to LR732085 [65].

The locations of the putative rRNA array were identified using BLAST searches with NCBI 18S sequences (ITS: MW251838; LSU: MW567773) as queries. Putative telomeres were identified based on manually searching for scaffold ends with multiple repeats of the sequence “CACTAA” or its reverse complement “TTAGTG”.

## Results

### v2.0 genome assembly

The PacBio data generated consisted of ~ 666,000 reads, with an average length of 7 kb and a total of 4.7 Gb of data. Based on the genome size estimate of 83.7 Mb [24], this indicated a coverage of 56x, which is consistent with the alignment depth of our PacBio reads (mean depth: 56.8). Our updated assembly of the *Termitomyces cryptogamus* genome consisted of 64 contigs with a total length 70 Mb (Table 1). Compared to the v1.0 reference assembly the N50 increased over 10-fold. One contig (scaffold_18) has significant similarity to a previous mitochondrial sequence [37]. Analysis of the BUSCO gene set revealed that 96.6% of expected Basidiomycota genes are present in the assembly, an increase over the v1.0 assembly (Table 1).

**Table 1:** Summary statistics of the *Termitomyces cryptogamus* v1.0 reference genome and the updated v2.0 assembly

	v1.0 Assembly [24]	V2.0 CANU Assembly	V2.0 Assembly polished with Pilon
QUAST Analysis			
Fragments	11,244	64	64
Length (Mb)	68.49	70.03	70.03
N50 (Mb)	0.262	2.721	2.721
BUSCO Analysis			
Complete	94.2%	96.3%	96.6%
Single	92.9%	94.7%	95.0%
Duplicated	1.3%	1.6%	1.6%
Fragmented	2.3%	1.7%	1.6%
Missing	3.5%	2.0%	1.8%
Predicted Genes			
Total	11,566	–	12,567
CAZymes	201^a^	–	386

^a^CAZyme predictions of v1.0 assembly are as reported previously and were not recalculated using current databases used for v2.0 annotations

Alignment of the v1.0 assembly to our assembly showed that every scaffold (> 200 bp) in the v1.0 assembly was present in our v2.0 assembly. Conversely, only the two smallest contigs from our v2.0 assembly were not present in the v1.0 assembly (Supplementary Fig. 2).

### Putative telomeres and ribosomal DNA

Manual inspection of the ends of the contigs of assembly v2.0 revealed a reoccurring 6 bp repeat, CACTAA or its reverse complement TTAGTG, at the ends of several contigs. We found this repeat on the ends of 15 of the contigs in our assembly. The sequence was repeated 13.4 times on average, the minimum repeat number was four and the maximum 21.

Manual searches of the assembly using the 5.8S, 28S and 18S ribosomal subunits as BLAST queries (NCBI: JAGYZC010000003.1) revealed that two small scaffolds (scaffold_41 and scaffold_33) contained only ribosomal repeats (two complete and four incomplete operons, respectively), and scaffold_3 contained one ribosomal operon at its start.

### Linkage mapping

To evaluate the effectiveness of our visual identification of homokaryons for GBS analysis, 37 single-basidiospore cultures were chosen based on growth rate and analysed using our RFLP marker: 12 suspected heterokaryons and 25 putative homokaryons (Supplementary Fig. 1). None of the putative homokaryons and roughly half of the suspected heterokaryons (5/12) showed heterozygosity at this locus. Only half of heterokaryons would be expected to be heterozygous for any given locus, as the other half would be homozygous for either of the parental alleles. These 5 confirmed heterokaryons along with all other suspected heterokaryons in the mapping population were excluded from the further analyses. From the remaining presumed homokaryons in the mapping population, 88 were chosen at random for GBS analysis. In addition, the parent heterokaryon was included in three replicates.

GBS yielded on average 2.9 million reads per sample, with a minimum of 0.8 million, and a maximum of 5.9 million reads. Variant calling of the combined GBS output and Illumina sequencing of mt50a yielded 1,019,581 markers. After filtering, a total of 1417 high-quality markers and 88 individuals from the mapping population were used for linkage mapping. The filters used removed the parental replicates as having excess heterozygosity.

Initial linkage mapping resulted in 13 linkage groups (LGs) with a total map length of 1101.3 cM (Fig. S2). In total 27 contigs from our v2.0 assembly were represented in the linkage map (Fig. S2, Table 2 and Supplementary Tables 2 and 3). The total length of the represented contigs was 61.4 Mb, which is 87.7% of the assembly. The sum of physical lengths between markers is 50.0 Mb, which is 71.4% of the total assembly. All markers from the same contig mapped to identical LGs except for two of the contigs from our assembly that were assigned to two different linkage groups (TIG015 and TIG049) (Supplementary Table 2). Both contigs have a large region without markers, i.e. 470 kb (TIG049) and 897 kb (TIG015) relative to the average marker distance (37 kb, s.d. 123 kb) that separates the two ends of each linkage group.

**Table 2:** Summary statistics of the strictly filtered linkage map and the forced order linkage map for *T. cryptogamus*

Strictly filtered data						Forced order							
Linkage Group	# of markers	Genetic length (cM)	Longest interval (cM)	Physical length (Mb)	Length between markers (Mb)	Linkage Group	# of markers	Genetic length (cM)	Longest inter-marker gap (cM)	Physical length (Mb)	Length between markers (Mb)	Start putative telomere	End putative telomere
**LG1**	117	66.9	19.7	3.97	2.48	**LG1**	167	71.6	18.2	3.98	3.35	Yes	Yes
**LG2**	154	149.8	34.6	8.02	7.08	**LG2**	217	156.8	21.6	8.02	7.08	No	Yes
**LG3**	74	41.4	14.6	4.76	4.56	**LG3b**	195	122.7	43.2	4.16	4.07	No	No
**LG4**	99	117.2	35.7	3.97	3.32	**LG4**	135	95.5	22.7	3.97	3.41	No	No
**LG5**	131	67.6	24.0	3.85	2.69	**LG5_LG11**	241	98.9	35.2	5.80	5.24	No	No
**LG6**	157	142.3	28.4	5.77	5.75	**LG6**	254	128.4	22.7	5.77	5.75	Yes	No
**LG7**	29	39.5	18.7	4.86	1.62	**LG7_LG8**	152	139.8	21.6	6.41	5.52	Ribo	Yes
**LG8**	93	77.9	24.9	1.55	2.95	**LG9**	202	108.0	18.2	5.32	4.89	Yes	No
**LG9**	174	126.9	24.8	5.32	4.81	**LG10_TIG058**	154	89.8	18.2	4.65	4.27	Yes	No
**LG10**	38	15.4	7.3	4.22	1.02	**LG12**	234	130.7	19.3	7.80	7.49	No	Yes
**LG11**	64	12.0	6.4	1.96	1.88	**LG13**	119	121.6	34.1	5.39	4.68	Yes	Yes
**LG12**	210	140.1	22.5	7.80	7.49	**LG14**	96	62.5	18.2	3.38	2.99	No	No
**LG13**	77	104.3	23.8	5.39	4.31	**LG15**	63	44.3	18.2	2.01	1.70	No	Yes
**Total:**	**1417**	**1101.3**		**61.44**	**49.96**	**Total:**	**2229**	**1370.4**		**66.66**	**60.44**		
**Average genome-wide recombination rate (cM/Mb)**^**a**^**:**					**22.0**	**Average genome-wide recombination rate (cM/Mb)**^**a**^**:**					**22.7**		

^a^Ratio of total genetic to total physical map-length indicates an average genome-wide recombination rate

### Forced order linkage map and recombination frequency

**Fig. 1:**
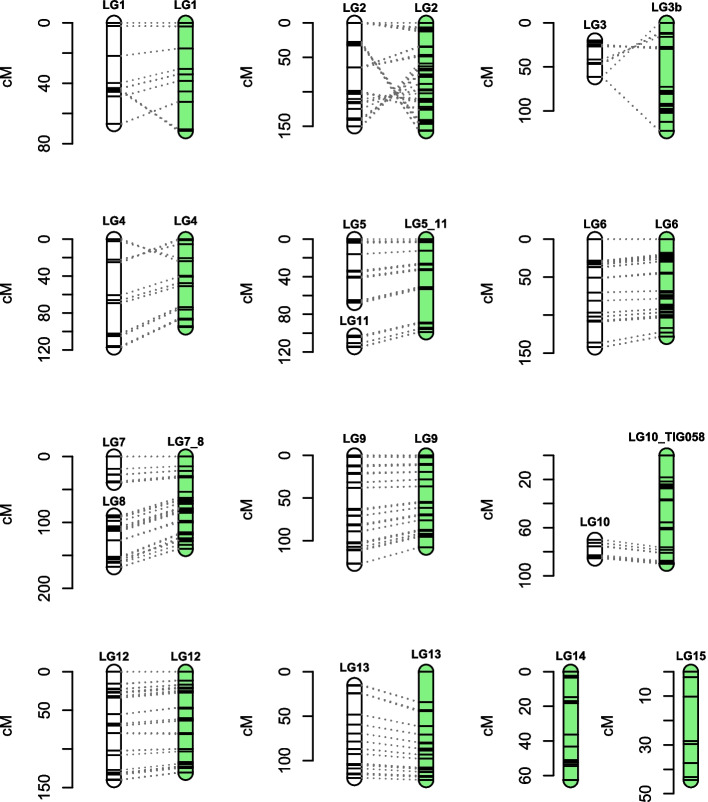
Comparison between linkage map (white) and forced order linkage map (green). Dotted lines indicate connection between identical markers

Our second mapping approach of calculating linkage between distal markers of the physical assembly yielded a total map length of 1370.4 cM (Table 2, Supplementary Fig. 3, Supplementary Tables 4 and 5). With this approach, we could include 30 contigs from the v2.0 assembly, three more than in the first mapping approach, adding up to 66.7 Mb, which is 95.2% of the total v2.0 assembly. Furthermore, the total physical length between markers increased to 60.4 Mb, which is 86.3% of the total v2.0 assembly. Comparison of both mapping approaches (Fig. 1 and Table 2) showed that for LG1, LG2, LG4, LG6, LG9 and LG12 the longest genetic interval between two markers decreased. Also, for LG4, LG6, LG9 and LG12 the total map length decreased, even though the added physical length between markers stayed the same or increased. For LG1 the map length increased by 7% whereas the added physical length between markers increased by 35%. The map length of LG2 increased by 5%, but the added physical length between markers stayed the same. For the combined linkage groups, LG5_LG11 and LG7_LG8, map length increased by 15 and 21%, whereas the added physical length between markers increased by 24 and 19% respectively. For LG10, LG14 and LG15 it is not possible to make a good comparison between mapping approaches as they were (largely) not present in the initial strictly filtered linkage map. Finally, for LG3 the genetic map length increased by almost 200%, yet the added physical length between markers decreased by 11% (Fig. 1).

**Fig. 2:**
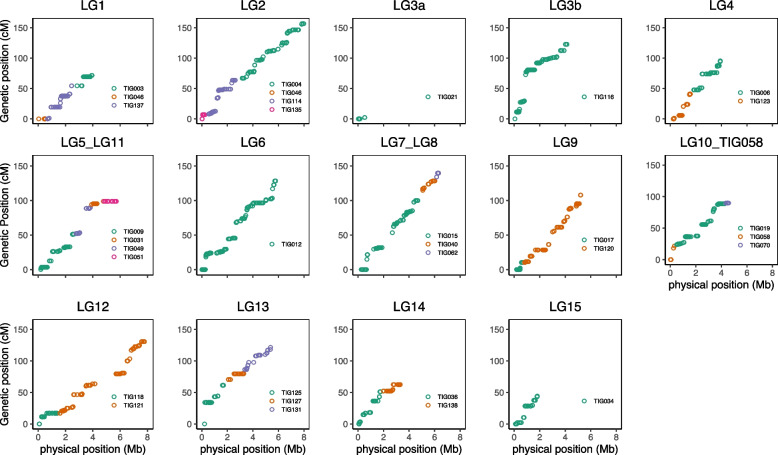
Plots of each linkage group in the forced-order linkage map with physical position of a marker as mapped to our assembly and on the y-axis the genetic position on the forced-order linkage map

**Fig. 3:**
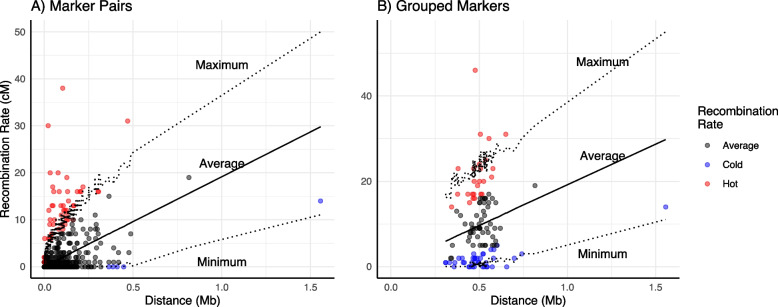
Number of crossovers (y-axis) plotted against marker distance between adjacent markers. **A** Genetic and physical distances between adjacent markers. **B** Physical and genetic distances averaged over intervals of approximately 500 kb. Red dots represent intervals with significantly more recombination then expected compared to the simulated datasets (*p* < 0.05), blue spots significantly less recombination. Lines give maximum, average, minimum recombination in simulated datasets

In a linkage map with many more markers than individuals it is expected that there are also many markers that are identical between individuals, i.e. that show exactly the same segregation pattern. There is, however, also the possibility that larger regions with no recombinant offspring are the result of uneven recombination rates across the genome. To examine this, we plotted the genetic distance against physical distance for our forced order map (Fig. 2). This revealed that for some contigs the genetic position increased in jumps rather than in a more continuous fashion, e.g. in LG1 and LG13. The largest jump was seen on LG3b, where a region of 0.1 Mb corresponded to a genetic distance of 43 cM. To test whether outlier distances in our genetic map deviate from an even distribution across the genome we compared the observed number of crossovers between two markers to a set of randomly sampled numbers of crossovers between those markers, under the assumption that physical marker distance is correlated to number of crossovers. In general, there was a positive relationship between physical distance between markers and the number of recombination events (Spearman’s correlation: 0.46; *p*-value < 2.2e-16). This analysis also showed that there are several outlier intervals that have significantly more or less recombination than others in all linkage groups (Fig. 3A+B). As marker distribution is not evenly spread across our assembly (Fig. 3A) we repeated the above-described test with our markers grouped in distances of 0.5 Mb (Fig. 3B), chosen as only 2.8% of marker pairs have distances larger than this. We found five intervals with less recombination than expected and 48 with more recombination than expected. The same test, but for grouped regions of approximately 500 kb, found 39 low-recombining and 25 high-recombining regions (Fig. 3B). When we compared the outcomes of the grouped and non-grouped marker distances, 4 low-recombining regions were present in both analyses, and 28 high-recombining regions (Supplementary Table 6).

### Localization of the mating type locus

**Fig. 4:**
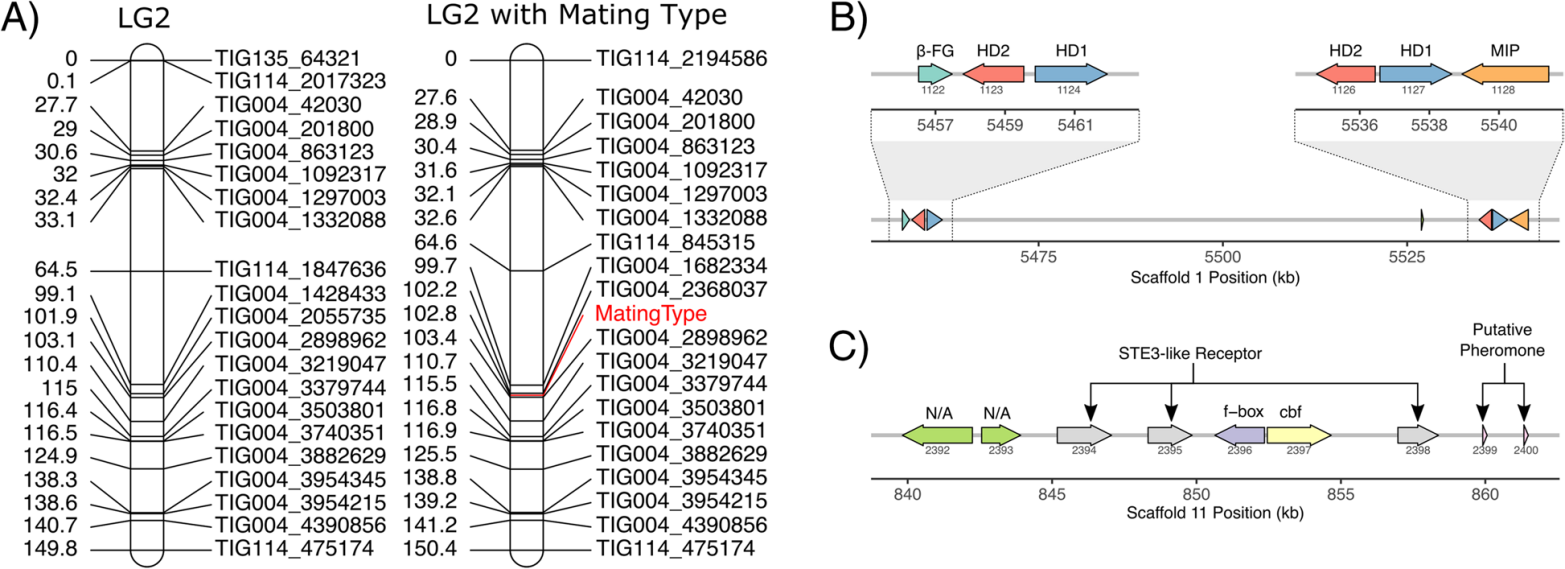
**A** LG2 without (left) and with Mating type with lines between identical markers. **B** Genes in HD mating type locus. **C** Genes in presumably monomorphic P/R locus identified through BLAST searches. Gene annotations in (**B**) assigned based on CDD matches

Crosses between 30 selected homokaryotic progeny indicate that the *T. cryptogamus* has a bipolar mating system, as roughly one half of the homokaryons were compatible with each tester (Supplementary Table 1). One mating type allele was found in 15 homokaryons and the other 15 had the alternative allele. The mating type mapped to the middle LG2 (with a LOD score of 7.5 and 8.7 with its neighbouring markers) and fit into the pre-existing map without significantly distorting or lengthening the map (Fig. 4A).

Comparison of the HD1 and HD2 genes from *Schizophyllum commune* to the v2.0 assembly confirmed that the HD locus of the mating type is found on scaffold_1 in the region predicted by the linkage map. The BLAST search results indicated two HD loci in this species. The b-Flanking gene was found at 5.457 Mb, while the Mitochondrial Intermediate Peptidase was found at 5.540 Mb, spanning an interval of ~ 100 kb [66]. Analysis of the coding sequences in this region identified two pairs of the HD1/HD2 pair with a large interior region devoid of coding sequences (Fig. 4B). While the PR locus does not appear to segregate in the offspring, since the bipolar variation is caused by the HD region, the genes for the pheromones and receptors are still present (Fig. 4C). Homologs of the Ste3 pheromone receptor were identified on scaffold_11, as well as two copies of putative pheromones nearby (Fig. 3B).

### Gene annotation

**Fig. 5:**
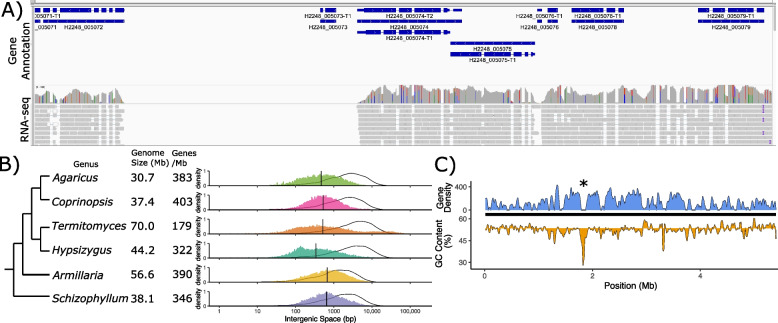
Gene spacing in *T. cryptogamus*. **A** Screenshot from IGV showing overlapping and closely space genes (blue bars), combined with large gene free regions. RNA-seq data is indicated below genes, showing that gene dense regions are almost completely transcribed. **B** Comparison of gene spacing in *T. cryptogamus* to other publicly available highly contiguous genomes. Black curve indicates simulated intergenic spacing given uniformly distributed genes. Coloured histogram indicates actual distribution of intergenic spaces (note log scale). Dotted vertical line indicates median intergenic space. **C** Blue shading above black line indicates gene density for scaffold_6 calculated across windows of 30 kb. Below black line indicates GC content, with the shading showing the difference from the genome wide average of 53%. Putative centromere location is indicated with an asterisk

A combined *de-novo* and evidence-based gene annotation resulted in the prediction of 12,567 genes. This was a slight increase of the previous annotation (Table 1). Many predicted genes of *T. cryptogamus* were very close to each other, often to the point of overlapping (Fig. 5A). Conversely, there were many regions of length > 1 kb that had no predicted genes. When compared to a hypothetical uniform distribution of genes (Fig. 5b: solid line), the intergenic spaces in *T. cryptogamus* had a somewhat bimodal distribution, one larger group of intergenic spaces much smaller than expected, and the other cluster larger than expected. Comparison to the relatively closely related *Hypsizygus marmoreus,* also a member of the Lyophyllaceae, genome shows a similar excess of smaller intergenic spaces, but without the excess of gaps larger than expected. Analysis of several other available annotated contiguous basidiomycete genomes shows these genomes all have smaller intergenic spacing than expected under uniform distribution, but that these genomes have a unimodal distribution, with a median intergenic space ~ 500 bp (Fig. 5B).

As fungal centromeres are often identified by a region of gene-free sequence > 20 kb, and an increased A/T%, we surveyed the *T. cryptogamus* genome for signs of centromeres. Some regions showed a correlated region of gene free space combined with increased A/T% (Fig. 5C), although when looking across the entire genome there was little consistent pattern. However, in many scaffolds there were several regions that were both gene-poor and A/T rich, more than the one centromere expected per chromosome (Supplementary Fig. 5).

## Discussion

### Genome assembly

In this study, we used long-read sequencing, the CANU assembler, and Pilon polishing with short reads to create a significantly more contiguous assembly of the genome of *Termitomyces cryptogamus.* As the BUSCO score of our v2.0 assembly even showed a slight improvement compared to the v1.0 assembly, improved contiguity of the genome did not come at the cost of the accuracy of single-gene annotations. The use of RNA-seq data to produce evidence-based annotations allowed us to disentangle many nearby genes, which had been fused into chimeric genes in the v1.0 assembly. Although we were not able to assemble full chromosomal sequences, the low number of scaffolds combined with the identification of 15 locations of telomeric repeats indicates that this assembly represents nearly complete chromosomal sequences.

From a technical perspective, it is important to note that in assessing the recombination landscape of the presently studied *Termitomyces* we assumed that the physical genome of the parents of our mapping population was identical to the individual that was sequenced for the reference assembly. In other words, we assumed that the physical position of the SNP markers was identical. This is a reasonable assumption as 1.) both samples originate from the same termite species, *M. natalensis*, which was shown to only cultivate a single biological species of *Termitomyces* [20, 35, 67], and, 2.) both samples were taken from nearby sampling locations, which means that they are likely to come from an interbreeding population. However, important differences can be observed that also influence our assessment of some regions of high and low recombination. For example, the alignment shows that on LG12, there is a region that is present in the reference assembly, but not in mt50a (Supplementary Fig. 4). This region coincides with a 1.5 Mb region without any markers, which means that the recombination analysis in this region is not reliable.

### Two mapping approaches

A common issue with generating dense SNP markers for linkage mapping is sites with many missing calls or low-coverage calls. To increase read depth per marker we used a “six-cutter” rather than the “five-cutter” often used for GBS [38]. This increased depth comes with the cost of a reduced number of markers since fewer cuts leads to fewer fragments of appropriate size for sequencing. With a reduced number of markers available, linkage mapping to scaffold a reference assembly relies on whether a less-frequent cutter will include smaller scaffolds. Although we could use only 0.14% of the raw SNPs, we managed to connect almost 90% of the assembly when strictly filtering the data. This shows that for linkage mapping a less frequent cutter is a potential option, although the decreasing cost of sequencing means that even use of a more frequent cutting site in GBS becomes more economical even at high depth. Bearing in mind the continuously reducing cost of sequencing, one could also consider low-coverage whole genome sequencing as an alternative for generating a linkage map [68]. In contrast to GBS, low-coverage whole genome sequencing is not dependent on the presence of a restriction site near the SNP in both parental genotypes. Low-coverage whole genome sequencing would produce more genotyping errors, but the low error rate of Illumina data (~ 0.01% of called bases) is outweighed by the increased number of markers. Recent studies have shown that mapping is practical even at depths as low as 0.01X [69, 70].

Including markers with many missing values or low coverage in a linkage map impacts the calculation of marker distance and may change marker order and distance. However, not including these markers also means discarding markers that do give reliable information. In addition to missing values, markers that have a very skewed allele frequency are also usually filtered out for linkage analysis. Skewed allele frequency may indicate erroneous calls and most mapping approaches assume Mendelian segregation ratios. However, in a biparental population such as ours, larger regions that show skewed segregation may be the result of segregation distortion, often the result of viability selection on element(s) in these regions [71].

To assess the impact of different marker filtering, we chose to take two mapping approaches. The first was to construct a linkage map using strictly filtered SNP data as a reliable basis for genetic mapping. In the second approach we filtered less strictly on missing data and skewed allele segregation, and visually inspected all markers at contig ends to see whether they were supported by surrounding markers. Subsequently, we calculated the extent of linkage between the first two and last two markers of each remaining contig. This approach allowed us to include, in addition to the more strictly filtered dataset, four contigs with a combined length of 5.8 Mb, i.e. more than 8% of the assembly.

In general, the two mapping approaches were highly congruent for the contigs that were included in both maps (Fig. 4). One of the most obvious differences between our two mapping approaches was LG3, which increased with almost 200% in genetic length, but decreased 11% in physical length. The use of the second mapping approach revealed that the most distal markers of TIG021 and TIG116 were not linked to each other with a LOD score higher than 5.5. Instead, the most distal marker of TIG021 was linked to the second marker of TIG116. Alignment of TIG021 against the whole reference assembly showed that the region with the markers of TIG021 that are linked to the second marker of TIG116, also aligns to the region in LG116 that follows the second marker. In other words, the linkage between TIG021 and TIG116 in our strictly filtered dataset is most likely an artefact of erroneous SNP calling due to a duplication between a region of TIG021 and TIG116.

Still, removal of TIG021 does not by itself explain the large increase in map length of LG3. On closer inspection a region of 3 Mb was filtered out in the strictly filtered map because of a skewed minor allele frequency (cumulative *p*-value lower than 0.01 under a binomial distribution).

### Segregation distortion

In addition to the region on LG3 with a very skewed allele frequency, we observed a significantly skewed allele frequency for more regions of the genome (Supplementary Table 7). This is presumably the reason that the contigs included in LG14 and LG15 were not present in the strictly filtered dataset. This highlights the effect on the genetic map of removing SNP markers with skewed allele frequencies; regions with segregation distortion will not be present in a linkage map.

In this context it is also interesting to note that despite extensive testing and repetition we were only able to obtain one of the two homokaryon genotypes of the parental heterokaryon, while we were able to repeatedly recover the other parental type (represented by mt50a). This may be due to lethal recessive mutations in the other, not recovered, parental haploid genome that prevents isolated growth. These mutations, and linkage to nearby markers, would result in a large region of segregation distortion. It has previously been shown that in long-term culture of *Schizophyllum commune,* mutations in the two nuclei of a heterokaryon can result in such defects that prevent recovery of both homokaryons [72]. Such recessive mutations are predictable in a heterokaryon, just as they would be in a diploid species. Further, evolution of a strain of *S. commune* found a case of a nucleus with a deleterious mutation that was compensated for by a mutation in the co-evolved nucleus [72]. It is not unlikely that a similar co-adaptation could be found in a *Termitomyces* heterokaryon, as these heterokaryons can grow vegetatively for multiple decades in a termite nest [17, 73].

### Mating type

*Termitomyces* fungi have been shown to lack clamp connections, which are the typical structures formed by heterokaryons of most basidiomycete species, and often used to assess mating success [74]. As it is currently not possible to artificially induce mushroom formation in this species, it is difficult to judge whether a mating is successful. However, it has been shown that a heterokaryon can be distinguished from a homokaryon based on phenotypic differences [35]. Our use of a RFLP marker allowed more certainty in phenotyping the offspring. As both mapping and searching the assembly for homologous sequences pointed to the same physical location in the genome this appears to be the mating-type region. In this region, we find two sets of the homeodomain genes (HD1/HD2), located closely together. This is found in other basidiomycetes, and in *Schizophyllum commune* even three sets of HD1 and HD2 loci and two additional HD1 genes were found [63].

Our mating tests indicate that *T. cryptogamus* has a bipolar mating type, with variation at only a single locus. Although *T. cryptogamus* has been reported to have a heterothallic mating system [75], it was unclear until now if this was a tetra- or bipolar system, whether variation exists at one or both mating type loci. It is thought that the tetrapolar mating system is the ancestral state in basidiomycetes [76, 77]. There are two main mechanisms to become bipolar: 1.) through physical linkage of the HD locus and the pheromone receptor locus, and, 2.) through the loss of the ability to recognize self from non-self in the pheromone/pheromone receptor locus through obtaining pheromone genes that fit one’s own receptor [78]. As we find a homolog of the yeast Ste3 receptor on a different linkage group than the HD locus, we hypothesize that this *Termitomyces* species became bipolar through the second mechanism, similar to what has been found in other species [76, 77]. This indicates that the pheromone/receptor pathway is likely still active, but now functions constitutively, instead of as a complementary pair between complementary individuals similar to what has been found in *Coprinellus disseminatus* [77].

### Karyotype of *Termitomyces*

One piece of the puzzle needed to create a truly finished assembly of *Termitomyces cryptogamus* is the karyotype, the number and shape of the chromosomes. In our assembly we could find 15 unique sequences with putative telomeres, which would mean that *Termitomyces* has at least eight chromosomes. Our forced linkage map contains 13 linkage groups, comprising 95.2% of the genome, which places a likely upper limit of 13 chromosomes. Thus, the *Termitomyces* genome likely contains between 8 and 13 chromosomes. This number of chromosomes is comparable to this number in other species of Agaricales. One method to quantify chromosome number is to localize the centromeres. With our improved annotation and transcriptome data, the localization of putative centromeres in our assembly may be possible based on gene-poor regions > 20 kb with low GC content [79]. However, our finding of frequent large (> 20 kb) intergenic regions of repetitive sequences makes the identification of centromeres more complicated.

Another approach often used to study karyotypes is Contour-clamped homogeneous electric field electrophoresis (CHEF). Despite extensive testing, we were not able to separate chromosome size bands (data now shown). It may be that some *Termitomyces* chromosomes are too large to be separated by CHEF. In the setup we used, chromosomes larger than ~ 8 Mb will not be separated, although chromosomes as large as 12.6 Mb have been separated [80]. As we found that *Termitomyces* likely has 8-13 chromosomes they will on average be between 6.4 and 10.5 Mb. So, in theory for some of the *Termitomyces* chromosomes it should be possible to separate them with CHEF, but for others it may not be possible. Another, more recent karyotyping method that has successfully been used on fungi is optical mapping which, particularly when combined with PacBio assemblies, can produce near-complete genome assemblies [81–83].

### Recombination analysis

It has been argued that species involved in a mutualistic interaction are under selection to have a lower rate of evolution [28]. In particular, there is empirical evidence that inhabitants in mutualistic symbioses have lower frequencies of sexual reproduction relative to the exhabitant, and relative to their non-mutualistic relatives, consistent with the idea that a benign internal environment favours lower rates of evolution (Law & Lewis, 1983). Arguing that *Termitomyces* is the inhabitant in the mutualism with fungus-growing termites and assuming that the recombination rate is one of the determinants of the rate of evolution, we hypothesised that *Termitomyces* would have a reduced recombination rate compared to free living species [27, 28]. We found that the average recombination rate for the *T. cryptogamus* to be between 17.9 cM/Mb and 22.6 cM/Mb. In fungi the mean recombination rate is 48.68 cM/Mb with a minimum of 1.4 cM/Mb and a maximum of 119.9 cM/Mb [84], which would indeed mean that the *Termitomyces* species we studied has a relatively low recombination frequency. However, this analysis included only 15 fungal species, of which four were basidiomycetes, and of which only two are in the same order (Agaricales). Additional recombination rates include: *Pleurotus ostreatus* 28.5 cM/Mb [85] and *Lentinula edodes* 18.4 cM/Mb [86], which is similar to the per Mb recombination rate that we observed. Recent analysis of the forest pathogen *Armillaria ostoyae* and the fairy-ring mushroom *Marasmius oreades* showed recombination rates of 17.9 and and 27.8 cM/Mb, respectively [65, 87]. These recent estimates used similar methodology to the present study, and recovered similar recombination rates per Mb of genome.

We can further question how much biological meaning the per Mb recombination rate has. Firstly, much of the recombination that occurs during meiosis is the sorting of parental chromosomes, independent of the rate of crossovers [29]. Using the $\overline{r}$ statistic recently introduced, segregation of the between 8 to 13 chromosomes of *T. cryptogamus* contribute approximately an order of magnitude more genetic diversity than crossovers [88]. However, any genome-wide measured recombination rate is also linked to the number of chromosomes an organism has; for correct segregation during meiosis at least one crossover needs to take place per pair of homologous chromosomes. Furthermore, genome expansion from repetitive sequences or other “junk” DNA will result in a reduction in cM/Mb recombination rate, although the recombination between coding genes is unchanged. As we see in *T. cryptogamus*, the genome size is larger than other Basidiomycetes included in genetic mapping studies. This could explain the reduced cM/Mb recombination rate. When only looking at map length, the recombination frequency we observed in *T. cryptogamus* is comparable to that of other Agaricales. Thus, although we do find a reduced rate of recombination in a cM/Mb measurement, the total map length is similar to free-living fungi, and thus our linkage map data are not consistent with the prediction that inhabitants in a mutualistic symbiosis are under selection for lower rates of evolution. However, the hypothesis specifically describes a lower rate of evolution, not necessarily related to the frequency of sexual reproduction or the recombination rate. A logical next step would be to determine if the rate of mutation, the fuel for evolution, is reduced in this species compared to free-living taxa.

## Conclusion

Understanding how and why recombination rate varies between taxa, species, sexes, individuals, and across the genome is a major challenge in biology. How recombination landscapes evolve and what impact this has on organisms deserves further attention. It remains unclear whether selection on the recombination landscapes affects the stability of mutualisms. We showed that *T. cryptogamus* harbours regions of both high and low recombination rates, which could in theory serve as targets of selection. This symbiotic fungus appears to have a relatively low per Mb recombination rate compared to other fungi. However, the absolute map length is comparable to other Agaricales, although comparative data is limited. The increasing rate at which contiguous genomes and linkage maps are being produced for various taxa will provide the necessary data to answer these questions.

## Supplementary Information

**Additional file 1: Supplementary Table 1.** Crossing table between 30 individuals from the mapping population against 6 siblings from the mapping population with legend. A “+” indicates a successful heterokaryon, “-“ no heterokaryon, “U” indicates unclear, and “NC” indicates cross not performed. **Supplementary Table 2.** An overview of the number of markers per contig present in each linkage group in the strictly filtered linkage map. **Supplementary Table 3.** Linkage group assignment and genetic position for each marker in the strictly filtered dataset. **Supplementary Table 4.** An overview of the number of markers per contig present in each linkage group in the Forced order linkage map. **Supplementary Table 5.** Linkage group assignment and genetic position for each marker in the Forced Order Linkage map. **Supplementary Table 6.** Intervals between markers and between grouped markers with less or more recombination than expected. **Supplementary Table 7.** Markers with a skewed minor allele frequency (cumulative *p*-value < 0.05 under binomial distribution).

**Additional file 2: Supplementary Figure 1.** Matings and genotyping of homo and heterokaryons. A) successful mating between individual 3 and 7. B) further clarification by transferring a piece of mycelium from both homokaryons (left and right) and the interaction zone of the mating (middle). C) RFLP analysis using NdeI restriction digest of PCR amplification of ef1-α on 12 suspected heterokaryons. D) RFLP analysis of parental heterokaryon. E) 25 presumed homokaryons using a marker for which the parent heterokaryon (top right) was heterozygous. Sizes of molecular ladder (lane M) are indicated on the left. The length of the undigested fragment targeted by PCR was 591 bp and digestion products of 417 bp and 173 bp. (Note that lower band of 173 bp is not visible).

**Additional file 3: Supplementary Figure 2.** A) Linkage map of strictly filtered data. Only unique markers are shown. B) Forced order linkage map.

**Additional file 4: Supplementary Figure 3.** DotPlotly visualisation of the alignment between the T. cryptogamus assembly v2.0 and the T. cryptogamus v1.0 assembly. On the x-axis the contigs of the v2.0 assembly, on the y-axis the scaffolds of the v1.0 assembly. Vertical and horizontal lines indicate edges of scaffolds or contigs. Dots indicate regions of similar sequence.

**Additional file 5: Supplementary Figure 4.** DotPlotly visualisation of the alignment between the contigs of the new reference assembly that form LG12 in the forced order linkage map and the assembly of mt50a. On the x-axis the contigs of the new reference genome, on the y-axis the scaffolds of the mt50a assembly.

**Additional file 6: Supplementary Figure 5.** Gene density and GC% across 15 largest scaffolds. Colours and values represented are as described in Fig. 5C.

**Additional file 7: Supplemental File 1 and Supplemental File 2.** Original, uncropped, gel electrophoresis images used for Supplementary Fig. 1.

## Data Availability

The updated v2.0 genome assembly is available as Genbank accession JAGYZC000000000. PacBio reads and GBS data are available under project PRJNA648478. RNA-seq data is available from SRR14421687 to SRR14421693. Living cultures are available upon request to corresponding author.
