# Supplementary material for: A genetic linkage map and improved genome assembly of the termite symbiont Termitomyces cryptogamus
Source: BMC Genomics. 2023 Mar 16;24:123. doi: 10.1186/s12864-023-09210-x (PMC10021994; doi:10.1186/s12864-023-09210-x)
Supplement: Supplementary file 1 — Additional file 1: Supplementary Table 1. Crossing table between 30 individuals from the mapping population against 6 siblings from the mapping population with legend. A “+” indicates a successful heterokaryon, “-“ no heterokaryon, “U” indicates unclear, and “NC” indicates cross not performed. Supplementary Table 2. An overview of the number of markers per contig present in each linkage group in the strictly filtered linkage map. Supplementary Table 3. Linkage group assignment and genetic position for each marker in the strictly filtered dataset. Supplementary Table 4. An overview of the number of markers per contig present in each linkage group in the Forced order linkage map. Supplementary Table 5. Linkage group assignment and genetic position for each marker in the Forced Order Linkage map. Supplementary Table 6. Intervals between markers and between grouped markers with less or more recombination than expected. Supplementary Table 7. Markers with a skewed minor allele frequency (cumulative p-value < 0.05 under binomial distribution). [file 12864_2023_9210_MOESM1_ESM.docx]

**Supplementary Table 1:** Crossing table between 30 individuals from the mapping population against 6 siblings from the mapping population with legend. A “+” indicates a successful heterokaryon, “-“ no heterokaryon, “U” indicates unclear, and “NC” indicates cross not performed.

| sibling | 3 | 6 | 19 | 32 | 49 | 60 |
| --- | --- | --- | --- | --- | --- | --- |
| 3 | NC | + | U | U | - | - |
| 6 | NC | NC | + | + | U | + |
| 19 | NC | NC | NC | - | - | - |
| 32 | NC | NC | NC | NC | - | - |
| 49 | NC | NC | NC | NC | NC | - |
| 60 | NC | NC | NC | NC | NC | NC |
| 7 | + | - | U | U | + | + |
| 8 | U | - | + | U | + | + |
| 12 | - | + | - | - | - | - |
| 14 | - | + | - | U | - | - |
| 15 | U | - | U | + | + | + |
| 20 | + | - | + | + | + | + |
| 21 | + | - | + | + | + | + |
| 22 | + | - | + | U | U | + |
| 23 | U | - | U | U | U | + |
| 24 | - | + | - | - | - | - |
| 26 | - | + | - | - | - | - |
| 29 | U | U | + | U | + | + |
| 31 | + | - | U | + | + | + |
| 34 | + | - | + | + | + | + |
| 35 | U | + | - | - | - | - |
| 36 | - | + | - | - | - | - |
| 38 | + | - | + | + | + | U |
| 42 | + | - | + | + | + | + |
| 43 | U | + | - | - | - | U |
| 47 | + | - | U | + | + | U |
| 48 | - | + | U | - | - | - |
| 51 | + | - | + | U | + | + |
| 55 | + | - | + | + | + | + |
| 58 | - | + | - | - | - | - |

**Supplementary Table 2:** An overview of the number of markers per contig present in each linkage group in the strictly filtered linkage map.

**Supplementary Table 3:** Linkage group assignment and genetic position for each marker in the strictly filtered dataset. Table can be found online: <https://doi.org/10.6084/m9.figshare.12387104.v1>

**Supplementary Table 4:** An overview of the number of markers per contig present in each linkage group in the Forced order linkage map.

**Supplementary Table 5:** Linkage group assignment and genetic position for each marker in the Forced Order Linkage map. Table can be found online: <https://doi.org/10.6084/m9.figshare.12387104.v1>

**Supplementary Table 6:** Intervals between markers and between grouped markers with less or more recombination than expected.

| Linkage Group | Ungrouped marker distances | | | | Grouped marker distances | | | |
| --- | --- | --- | --- | --- | --- | --- | --- | --- |
|  | marker1 | marker2 | more/less | Number of crossovers | marker1 | marker2 | more/less | Number of crossovers |
| LG1 | TIG046_36278 | TIG046_446912 | less | 0 | TIG046_36278 | TIG137_101668 | less | 0 |
|  | TIG137_194951 | TIG137_326862 | more | 16 | TIG137_101668 | TIG137_616681 | more | 17 |
|  |  |  |  |  | TIG137_1153062 | TIG137_1673212 | less | 3 |
|  |  |  |  |  | TIG003_948604 | TIG003_1352904 | less | 2 |
| LG2 | TIG114_2017323 | TIG114_1889693 | more | 19 | TIG114_2280117 | TIG114_1774042 | more | 31 |
|  | TIG114_1807601 | TIG114_1774042 | more | 10 |  |  |  |  |
|  |  |  |  |  | TIG114_1774042 | TIG114_1287489 | less | 2 |
|  | TIG004_3933782 | TIG004_3882694 | more | 9 | TIG004_3954345 | TIG004_3445640 | more | 17 |
|  |  |  |  |  | TIG004_2915688 | TIG004_2440748 | less | 2 |
|  |  |  |  |  | TIG004_2440748 | TIG004_2055735 | less | 2 |
|  | TIG004_1376277 | TIG004_1332088 | more | 13 | TIG004_1554430 | TIG004_1092317 | more | 17 |
|  |  |  |  |  | TIG004_1092317 | TIG004_511900 | less | 2 |
| LG3b | TIG116_62089 | TIG116_187305 | more | 10 | TIG116_62089 | TIG116_561998 | more | 24 |
|  | TIG116_417696 | TIG116_475881 | more | 10 |  |  |  |  |
|  | TIG116_796442 | TIG116_900736 | more | 38 | TIG116_561998 | TIG116_1038617 | more | 46 |
|  |  |  |  |  | TIG116_1038617 | TIG116_1514483 | less | 1 |
|  |  |  |  |  | TIG116_2545470 | TIG116_3068260 | less | 3 |
| LG4 | TIG006_756615 | TIG006_790338 | more | 20 | TIG006_338148 | TIG006_807307 | more | 23 |
|  |  |  |  |  | TIG006_807307 | TIG006_1269618 | less | 0 |
|  |  |  |  |  | TIG006_1269618 | TIG006_1734730 | less | 2 |
|  | TIG006_1935223 | TIG006_1966682 | more | 9 | TIG006_1734730 | TIG006_2203718 | more | 17 |
|  | TIG006_2203707 | TIG006_2203711 | more | 1 |  |  |  |  |
| LG5_LG11 |  |  |  |  | TIG009_184523 | TIG009_653362 | less | 3 |
|  | TIG009_941471 | TIG009_1051621 | more | 12 | TIG009_653362 | TIG009_1124869 | more | 20 |
|  |  |  |  |  | TIG009_1124869 | TIG009_1583737 | less | 1 |
|  | TIG009_2312253 | TIG009_2513712 | more | 16 | TIG009_2048611 | TIG009_2513712 | more | 16 |
|  |  |  |  |  | TIG009_2513712 | TIG049_796701 | less | 1 |
|  | TIG049_796701 | TIG049_327036 | more | 31 | TIG049_796701 | TIG049_146598 | more | 31 |
|  |  |  |  |  | TIG049_146598 | TIG031_524790 | less | 2 |
|  |  |  |  |  | TIG031_524790 | TIG051_244525 | less | 3 |
|  |  |  |  |  | TIG051_244525 | TIG051_899834 | less | 0 |
| LG6 | TIG012_262603 | TIG012 | more | 16 | TIG012_18068 | TIG012_587832 | more | 21 |
|  | TIG012_316378 | TIG012 | more | 2 |  |  |  |  |
|  | TIG012_713865 | TIG012 | less | 0 | TIG012_587832 | TIG012_1161201 | less | 0 |
|  |  |  |  |  | TIG012_1161201 | TIG012_1636398 | less | 2 |
|  | TIG012_1907276 | TIG012 | more | 13 | TIG012_1636398 | TIG012_2110103 | more | 16 |
|  |  |  |  |  | TIG012_2110103 | TIG012_2606729 | less | 1 |
|  | TIG012_2606729 | TIG012 | more | 20 | TIG012_2606729 | TIG012_3141516 | more | 25 |
|  |  |  |  |  | TIG012_4242271 | TIG012_4784825 | less | 0 |
|  |  |  |  |  | TIG012_4784825 | TIG012_5385813 | less | 5 |
|  | TIG012_5512521 | TIG012 | more | 12 | TIG012_5385813 | TIG012_5766514 | more | 23 |
| LG7_LG11 | TIG015_650434 | TIG015 | more | 13 | TIG015_253572 | TIG015_743544 | more | 19 |
|  | TIG015_700326 | TIG015 | more | 6 |  |  |  |  |
|  |  |  |  |  | TIG015_1233523 | TIG015_1565655 | less | 1 |
|  |  |  |  |  | TIG015_1565655 | TIG015_1875114 | less | 1 |
| LG9 | TIG120_1065147 | TIG120 | less | 0 | TIG120_994955 | TIG120_1440488 | less | 0 |
|  | TIG120_1902611 | TIG120 | more | 16 | TIG120_1886174 | TIG120_2322183 | more | 17 |
|  | TIG120_3364088 | TIG120 | more | 10 | TIG120_2919979 | TIG120_3497138 | more | 23 |
|  |  |  |  |  | TIG120_3497138 | TIG120_4062718 | less | 4 |
|  | TIG120_4289736 | TIG120 | more | 11 | TIG120_4062718 | TIG120_4406861 | more | 14 |
| LG10_TIG058 | TIG058_48307 | TIG058_255214 | more | 16 | TIG058_20151 | TIG019_294390 | more | 20 |
|  |  |  |  |  | TIG019_750661 | TIG019_1192793 | less | 0 |
|  |  |  |  |  | TIG019_1192793 | TIG019_1731291 | less | 1 |
|  | TIG019_1731291 | TIG019_2036560 | more | 16 | TIG019_2766571 | TIG019_3273747 | more | 23 |
|  | TIG019_2766571 | TIG019_2930038 | more | 13 |  |  |  |  |
|  |  |  |  |  | TIG019_3273747 | TIG019_3683765 | less | 1 |
|  |  |  |  |  | TIG019_3683765 | TIG070_83792 | less | 0 |
| LG12 |  |  |  |  | TIG118_637090 | TIG118_1181911 | less | 0 |
|  |  |  |  |  | TIG118_1181911 | TIG121_5841496 | less | 4 |
|  |  |  |  |  | TIG121_4309411 | TIG121_3998402 | less | 1 |
|  | TIG121_3654849 | TIG121_2098036 | less | 14 | TIG121_3654849 | TIG121_2098036 | less | 14 |
|  |  |  |  |  | TIG121_2098036 | TIG121_1601152 | less | 1 |
|  | TIG121_1505784 | TIG121_1288112 | more | 17 | TIG121_1601152 | TIG121_1100097 | more | 20 |
|  | TIG121_1100097 | TIG121_983108 | more | 12 | TIG121_1100097 | TIG121_721963 | more | 17 |
| LG13 | TIG125_213170 | TIG125_235335 | more | 30 | TIG125_213170 | TIG125_768008 | more | 30 |
|  |  |  |  |  | TIG127_706290 | TIG127_1233915 | less | 0 |
|  |  |  |  |  | TIG131_961716 | TIG131_1659774 | less | 2 |
| LG14 |  |  |  |  | TIG036_1390453 | TIG036_974191 | less | 1 |
|  |  |  |  |  | TIG138_866158 | TIG138_1337531 | less | 0 |

**Supplementary Table 7:** Markers with a skewed minor allele frequency (cumulative p-value < 0.05 under binomial distribution). Table can be found online: <https://doi.org/10.6084/m9.figshare.12387104.v1>
