# Supplementary figures and images for: A genetic linkage map and improved genome assembly of the termite symbiont Termitomyces cryptogamus
Source: BMC Genomics. 2023 Mar 16;24:123. doi: 10.1186/s12864-023-09210-x (PMC10021994; doi:10.1186/s12864-023-09210-x)

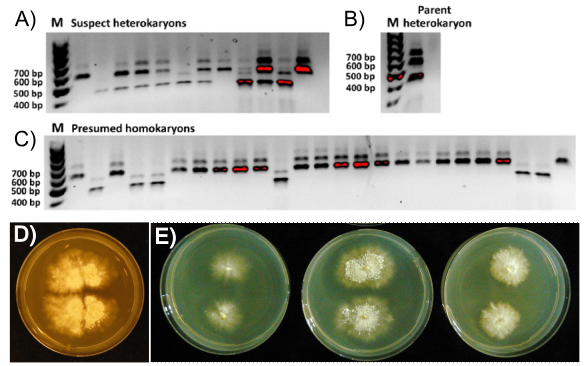

Supplement: Supplementary file 2 — Additional file 2: Supplementary Figure 1. Matings and genotyping of homo and heterokaryons. A) successful mating between individual 3 and 7. B) further clarification by transferring a piece of mycelium from both homokaryons (left and right) and the interaction zone of the mating (middle). C) RFLP analysis using NdeI restriction digest of PCR amplification of ef1-α on 12 suspected heterokaryons. D) RFLP analysis of parental heterokaryon. E) 25 presumed homokaryons using a marker for which the parent heterokaryon (top right) was heterozygous. Sizes of molecular ladder (lane M) are indicated on the left. The length of the undigested fragment targeted by PCR was 591 bp and digestion products of 417 bp and 173 bp. (Note that lower band of 173 bp is not visible). [file 12864_2023_9210_MOESM2_ESM.png]

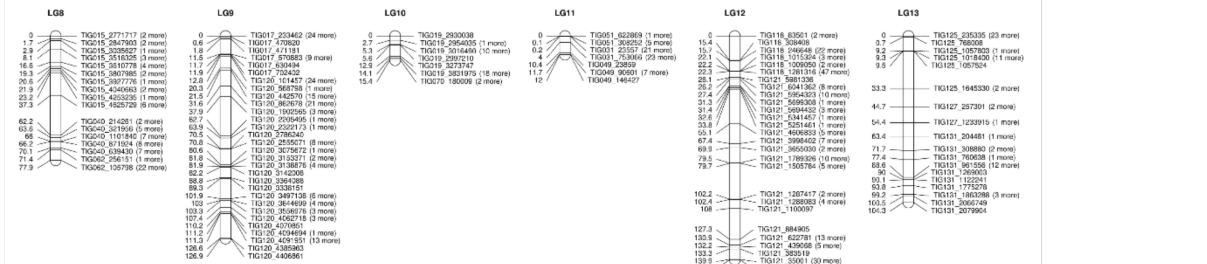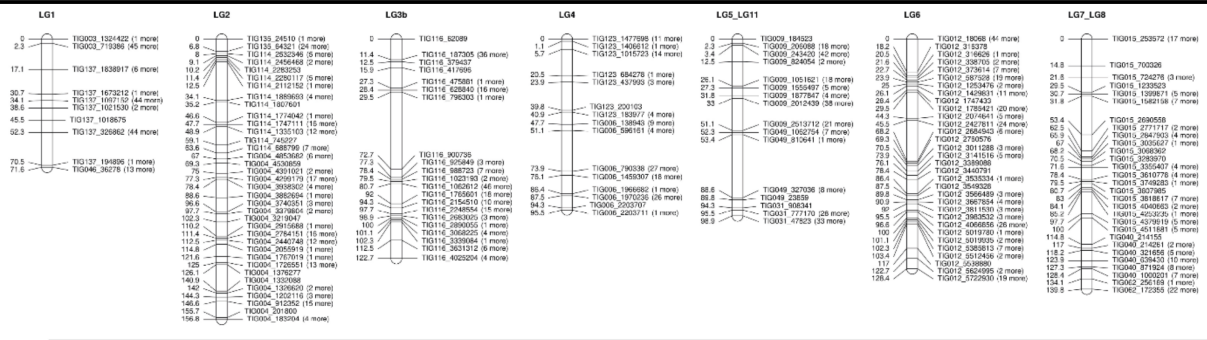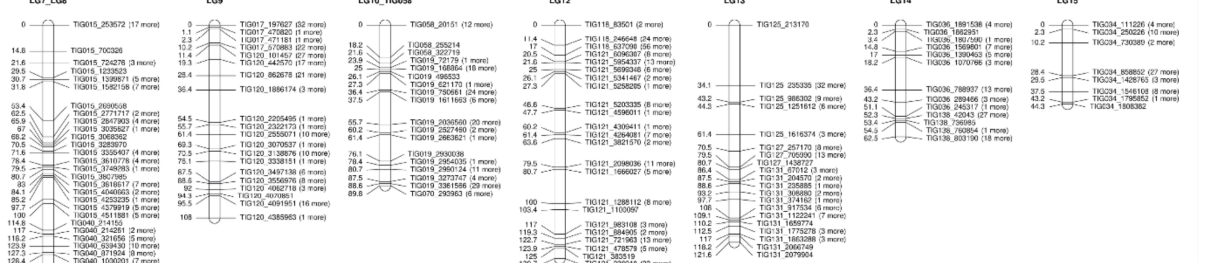

Supplement: Supplementary file 3 — Additional file 3: Supplementary Figure 2. A) Linkage map of strictly filtered data. Only unique markers are shown. B) Forced order linkage map. [file 12864_2023_9210_MOESM3_ESM.pdf]

```

alignment length (-m): 1000
minimum query aggregate alignment length (-q): 1000

```

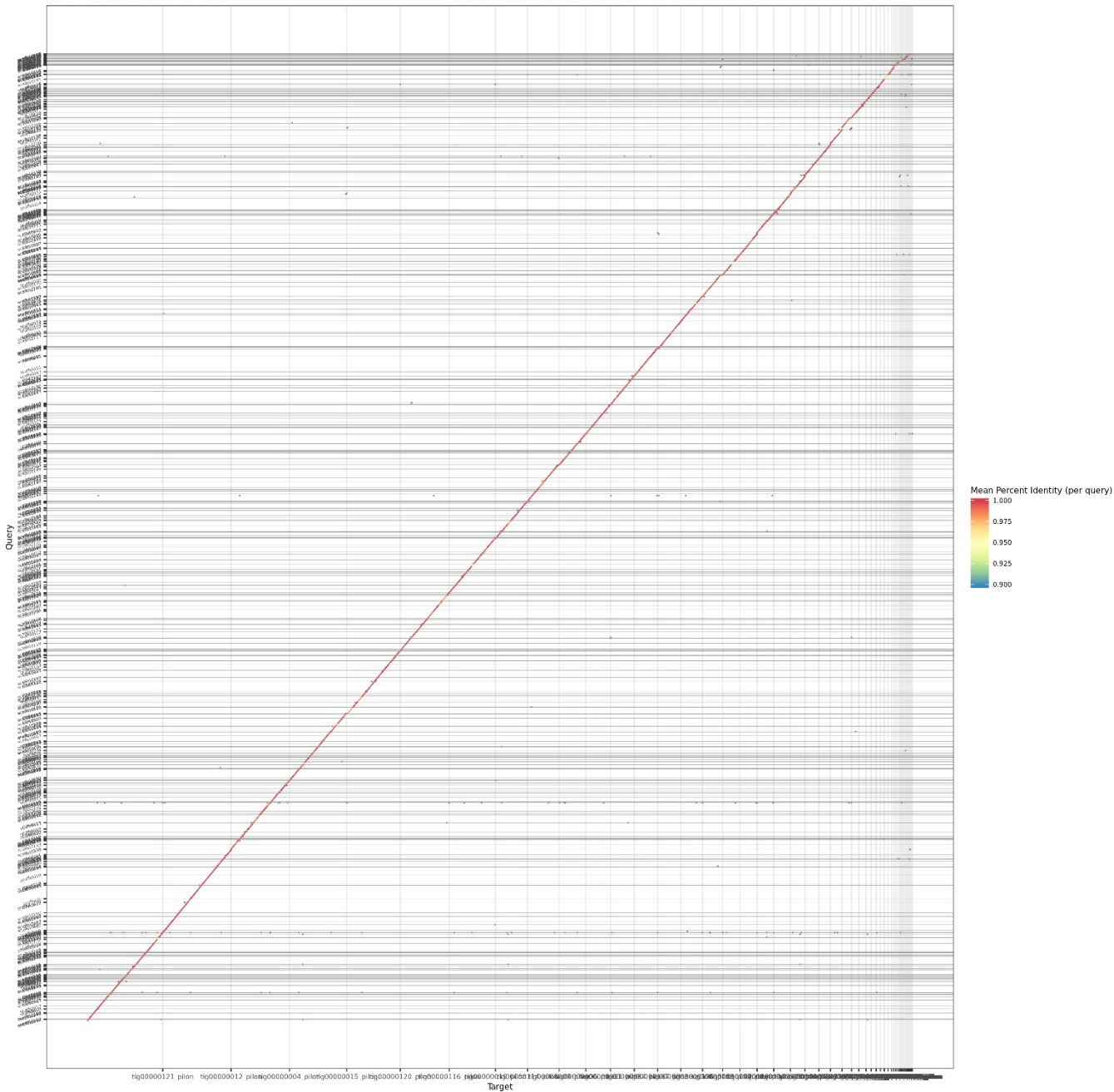

Supplement: Supplementary file 4 — Additional file 4: Supplementary Figure 3. DotPlotly visualisation of the alignment between the T. cryptogamus assembly v2.0 and the T. cryptogamus v1.0 assembly. On the x-axis the contigs of the v2.0 assembly, on the y-axis the scaffolds of the v1.0 assembly. Vertical and horizontal lines indicate edges of scaffolds or contigs. Dots indicate regions of similar sequence. [file 12864_2023_9210_MOESM4_ESM.pdf]

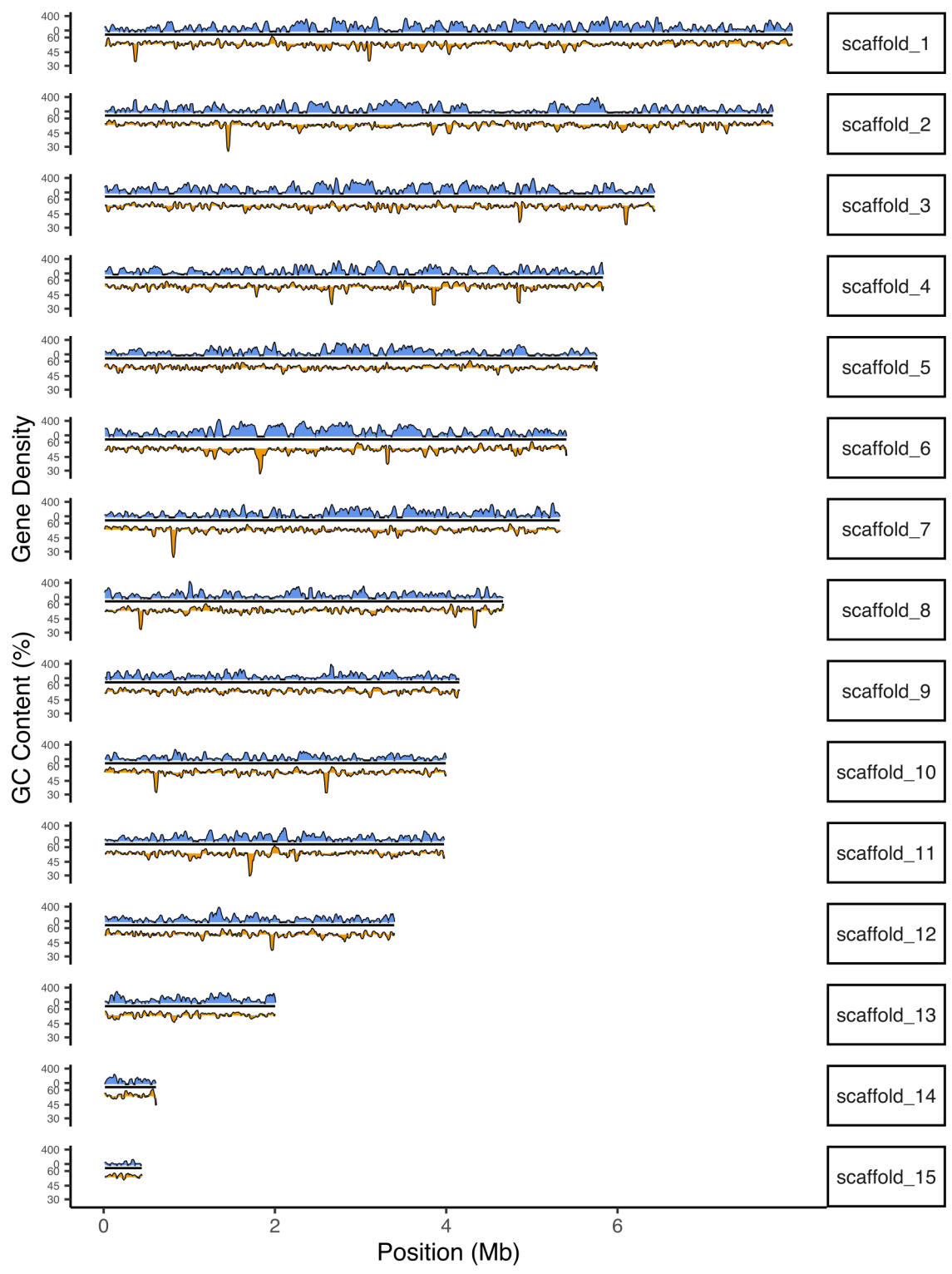

Supplement: Supplementary file 6 — Additional file 6: Supplementary Figure 5. Gene density and GC% across 15 largest scaffolds. Colours and values represented are as described in Fig. 5C. [file 12864_2023_9210_MOESM6_ESM.pdf]

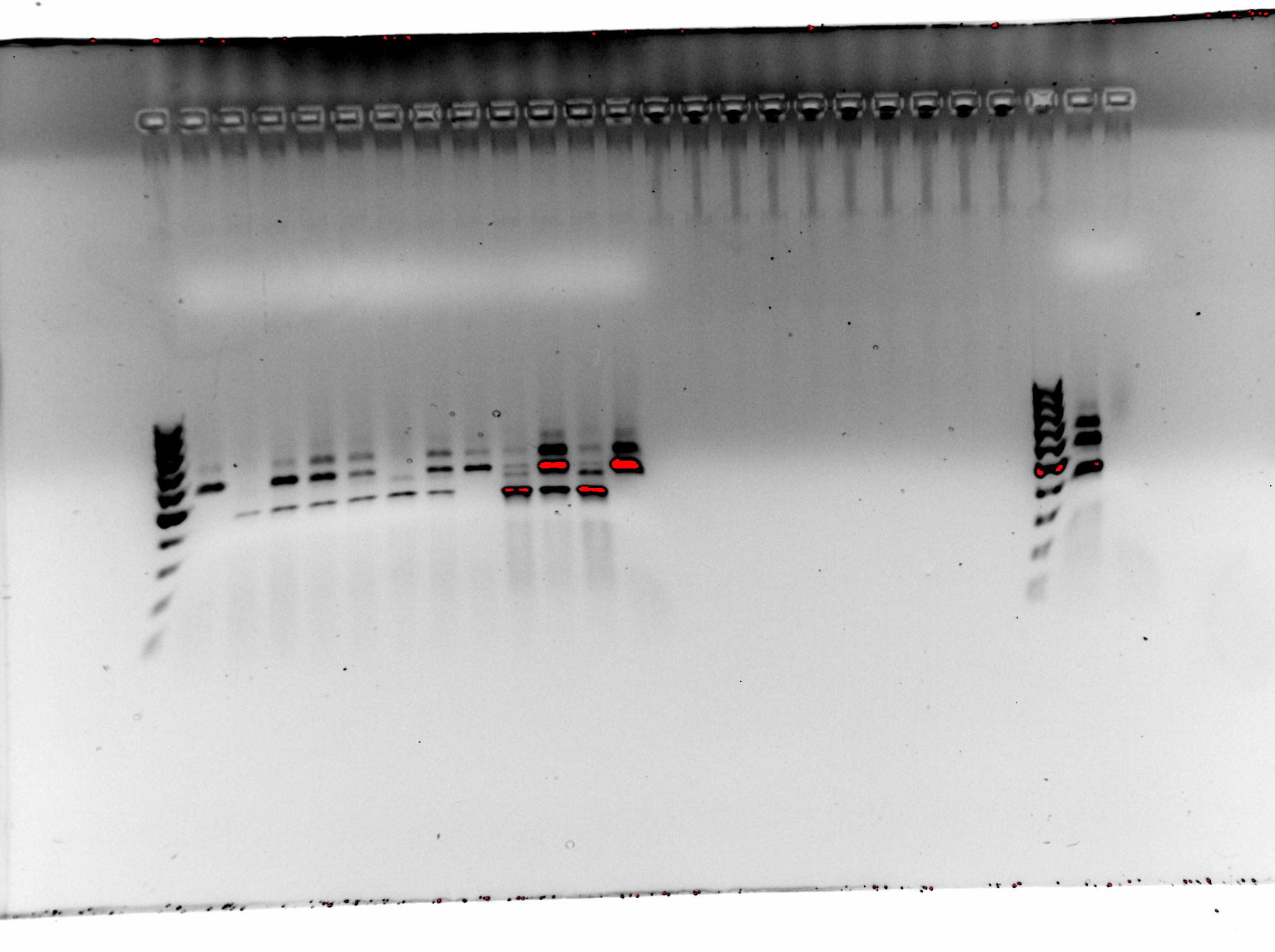

Supplement: Supplementary file 7 — Additional file 7: Supplemental File 1 and Supplemental File 2. Original, uncropped, gel electrophoresis images used for Supplementary Fig. 1. [file 12864_2023_9210_MOESM7_ESM.zip › Termitomyces EF sequence Digest3.1.tif]

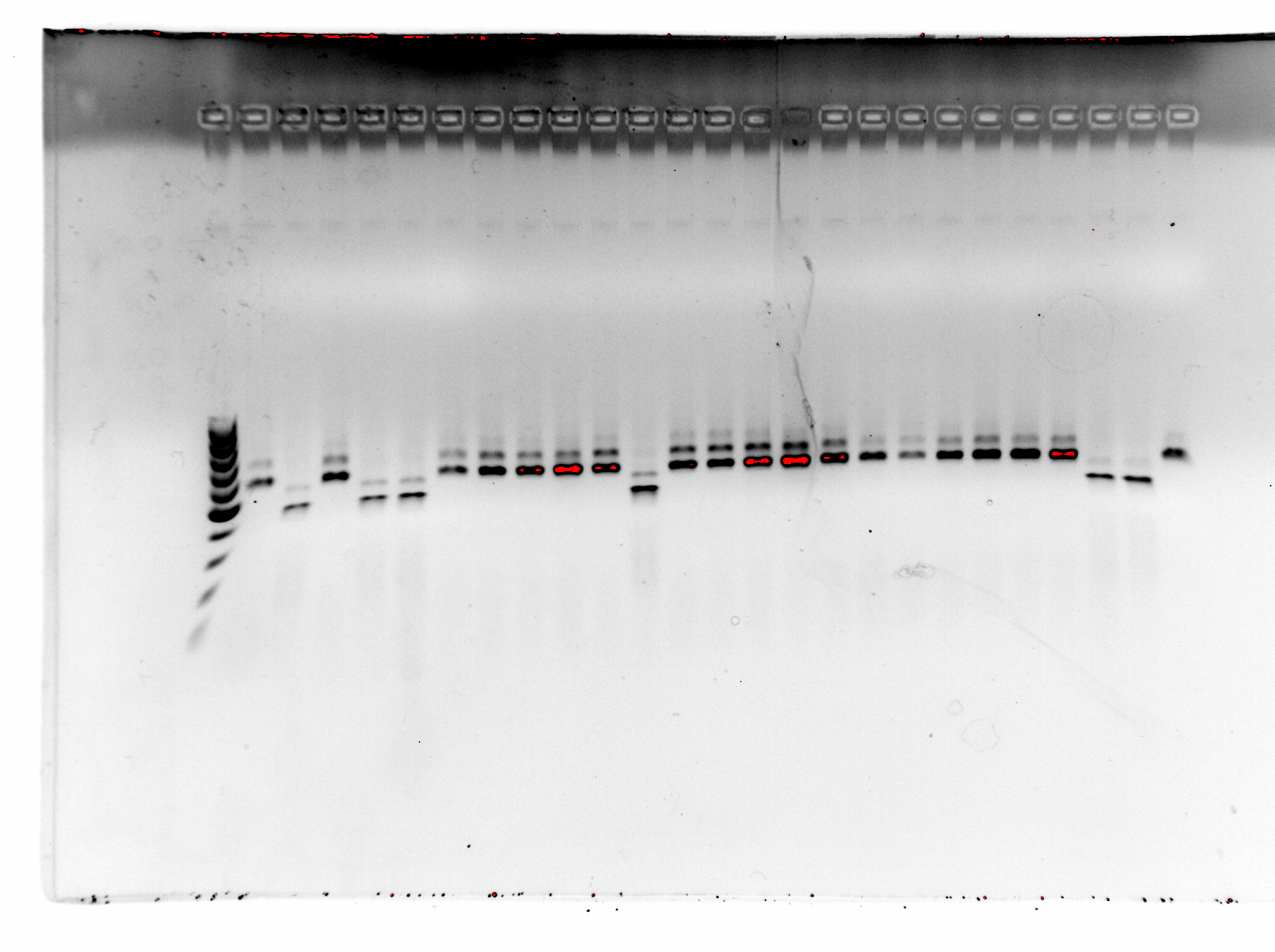

Supplement: Supplementary file 7 — Additional file 7: Supplemental File 1 and Supplemental File 2. Original, uncropped, gel electrophoresis images used for Supplementary Fig. 1. [file 12864_2023_9210_MOESM7_ESM.zip › Termitomyces EF sequence Digest3.2.tif]
