# Supplementary material for: A genetic linkage map and improved genome assembly of the termite symbiont Termitomyces cryptogamus
Source: BMC Genomics. 2023 Mar 16;24:123. doi: 10.1186/s12864-023-09210-x (PMC10021994; doi:10.1186/s12864-023-09210-x)
Supplement: Supplementary file 5 — Additional file 5: Supplementary Figure 4. DotPlotly visualisation of the alignment between the contigs of the new reference assembly that form LG12 in the forced order linkage map and the assembly of mt50a. On the x-axis the contigs of the new reference genome, on the y-axis the scaffolds of the mt50a assembly. [file 12864_2023_9210_MOESM5_ESM.pdf]

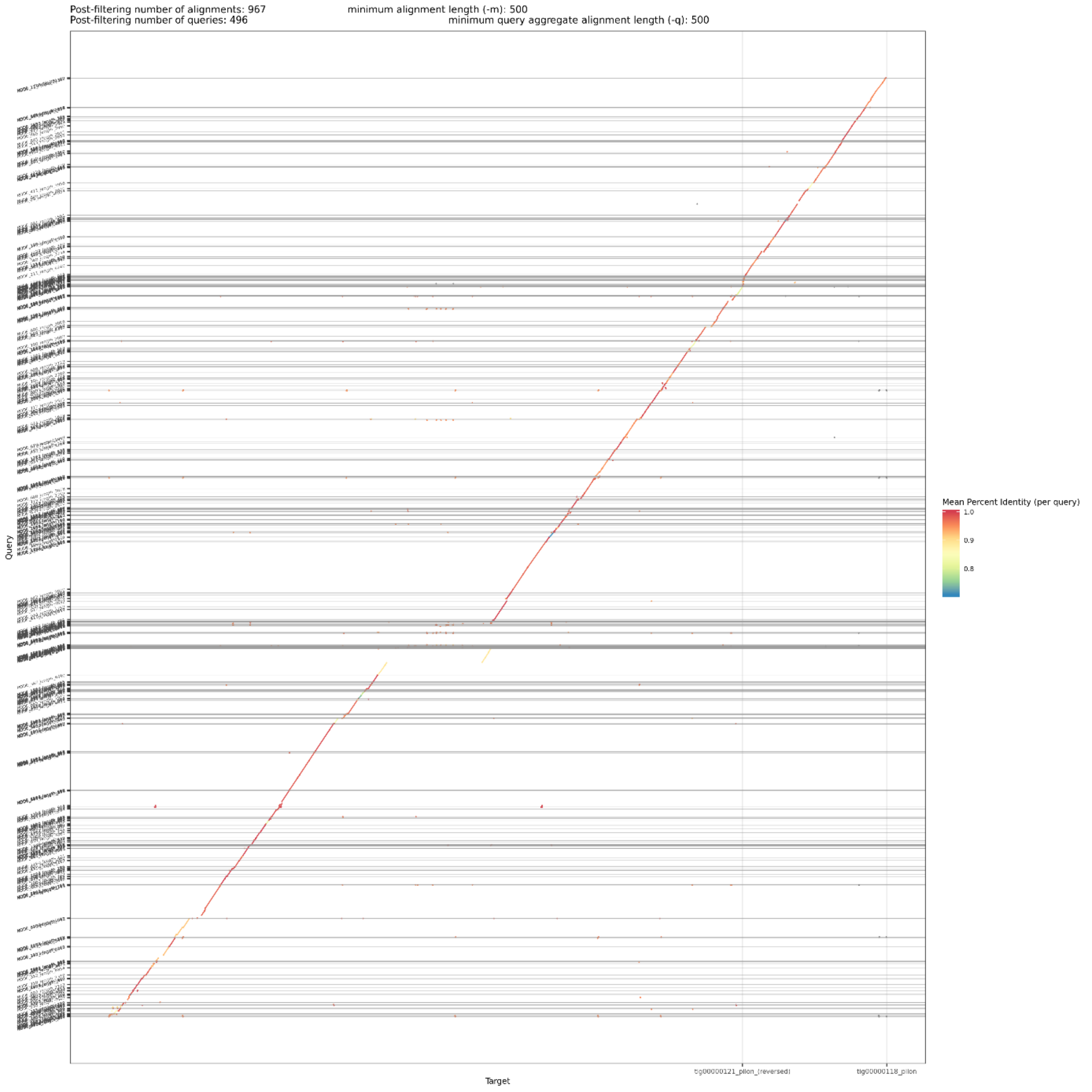

Post-filtering number of alignments: 967
Post-filtering number of queries: 496
minimum alignment length (-m): 500
minimum query aggregate alignment length (-q): 500
Query
Target
tig00000121_pilon_(reversed)
tig00000118_pilon
Mean Percent Identity (per query)
1.0
0.9
0.8
